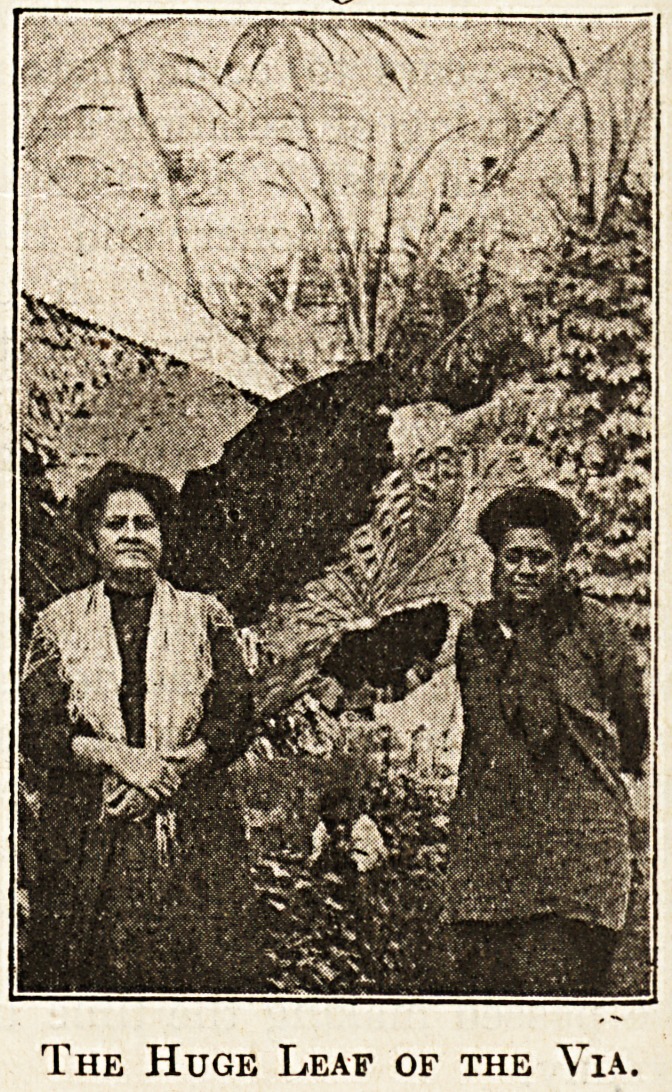# Curiosities of Native Treatment in Fiji

**Published:** 1919-01-18

**Authors:** T. R. St.-Johnston

**Affiliations:** Colonial Civil Service.


### CURIOSITIES OF NATIVE TREATMENT IN FIJI.

By T. R. ST.-JOHNSTON, M.R.C.S., L.R.C.P., Colonial Civil Service.

### MEDICAL.

Canoes, coral reefs, and cannibals! So, in an alliterative way, I have often heard Fiji spoken of. It is an extraordinary thing how few people really know anything about this island colony of the Antipodes, a colony that is growing in riches and importance every year, and whose tropical products are of the greatest value to the Empire. When the world has become sane once more the sons of Britain will be turning their feet once again to distant lands, just as the spirit of adventure has always moved our race; but with the difference, I think, that this war will have taught them the advantage, nay, the paramount necessity, of concentrating their manhood and strength in some corner of the earth over which floats the British flag.

There are probably few places in the world so far removed from London as Fiji, but wireless and air-ships have annihilated space for this generation; and in years to come distance will have none of the meaning of isolation and discomfort that it has had in the past. Fiji is almost exactly under the feet of the Londoner, and the future colonists now playing at "savages" in the nursery might be told without much exaggeration that if they were to dig straight down they would probably come out at Fiji, as we are roughly about the same meridian of longitude, and when it is 7 p.m. on Monday here, it is 7 a.m. on Tuesday out there. This, again, opens up a question of days of the week, and I have a friend in a neighbouring island who is able to sleep with his head in Tuesday and his feet in Monday, as the meridian runs right through his house. This sort of thing led to all kinds of evasions as to Sunday trading, liquor selling after hours, etc., so that a special law had to be passed making the time universal for the whole colony.

Fiji is the only British Crown Colony in the Pacific Ocean, though there are several British Protectorates there. It is a colony of considerable importance nowadays, due to its large exports of tropical produce, notably sugar, copra, and green fruit; and also to its political domination of the Pacific. But owing to the fact that most of its trade is with Australia, Canada, and New Zealand —the countries nearest to it—it is very little known in this part of the world. It is even said that not so very long ago a much-travelled and re-directed letter arrived in Fiji, bearing, among other inscriptions, one from the G.P.O. of London directing their forwarding branch to "try West Africa." This may or may not be true, but I *do* think that after this war even Fiji will be better known to all; for three separate contingents of whites, mostly planters and men of means, have given up everything and come over here, and a very large percentage of them have made the final sacrifice for their country; a small contingent of the natives, selected from the thousands who volunteered, are in France at present, doing special work; while those who are left, both whites and natives, have given freely of their cash and labour, with a result that some £50,000 in money, and shiploads of clothing and stores, have been forwarded to the Red Cross; and motor ambulances have been purchased and are being kept up in France by native contributions; for the loyalty and patriotism of this colony know no bounds.

But although Fiji as a colony is a very up-to-date one, yet the natives themselves have not progressed in all directions as much as they might have done. There are several reasons for this, but the chief one is undoubtedly the old primitive law of custom, which binds these South Sea natives in iron chains as nothing else does. Any original native customs that are good for their welfare have been wisely upheld by the Government as far as pos-

The Doctor Going his Rounds.

sible, but others—the more deleterious ones—are only now beginning to be dispersed before the advancing tide of education. And some of the native prejudices that have been hardest to fight have been those against the white man's ideas of medical treatment.

Now, medicine and surgery are sciences that unfortunately admit of very little compromise. Those of us who fully realise the depth of native prejudices would gladly meet the natives' point of view if it were possible, but when we see a horrible running ulcer left, native fashion, with only a dressing of a few filthy rags, or when we find a man with dysentery feeding on crabs and unripe fruit, and going from bad to worse, we feel that it is time to step in and intervene. Every year now one is glad to see that more progress is being made in medical treatment among the Fijians. Native medical students are trained at the Central Hospital and sent out, when qualified, to work among their own people; and gradually the islanders are

**Curiosities of Native Treatment in Fiji**—(*continued*).

beginning to recognise the advantages of modern science. But their own primitive ideas of medicine and surgery still linger in the more out-of-the-way places, and during eleven years' residence among them, partly as a doctor, I have endeavoured to collect some notes on the native methods as practised, and drugs as used, before the new generation have quite lost these ancient arts of their fathers.

Some of the native medicines are undoubtedly of value, but the scientific objection to them was that there was no definite system of dosage. A native "vuniwai" or doctor would take a handful of leaves of a certain plant, macerate them and make an infusion, and then give the drink (of unknown strength and varying dilution) indiscriminately to young or old, weak or strong; so that—although by the knowledge of centuries it might be known that that particular plant would cure that particular ailment—it was largely a matter of luck as to whether he gave a poisonous overdose or not. Moreover, it was also still more a matter of luck whether his diagnosis of the ailment to be treated was correct or not. Therefore it is no small wonder that the scientific white man has impatiently brushed aside all these native remedies as useless. But if the plants used by the natives, and the infusions made from them, were carefully subjected to analysis, it might well be found that valuable new remedies were lying hidden in that far-off colony in the South Seas; and the doctor going his rounds, often on an outrigger canoe, might be surprised to learn that drugs as potent as any in his travelling-case were lying at hand in the bush on all sides.

THE UPSTANDING HAIR OF THE NATIVE.

Among the native drugs used for anodyne effects are the "Vulokaka" leaves (*Vitex trifoliata*), a small portion of the leaf chewed up being inserted into a hollow, aching tooth with good results. As a matter of fact decayed teeth are very rare occurrences among the flashing ivories of these people, so that this treatment is not often required. Tonga-bark (*waro*) is another anodyne much used, and is now even finding a small market in America.

A favourite febrifuge is a decoction of leaves of the "Bovo," a small tree with a curiously blended green and white foliage. This to a certain extent takes the place of quinine, even among white settlers in out-of-the-way parts, though where quinine can be obtained the natives have learned its superior qualities and readily ask for it. At the time of the historic measles epidemic which swept away a third of the Fijian race, and before many white men with recognised drugs had invaded all parts of the colony, the natives would have done well if they had kept to their native febrifuges only; but—recklessly striving to get cool at any cost, and despite all warnings from the few medical men then in the colony—they used to lie down in streams to cool themselves, with pneumonia and death as a result in thousands of cases.

A native variety of sarsaparilla (a species of *smilax*) has long been used by the people, who fully appreciate its value. They chew the leaves and infuse them, and drink the strained liquor. For a cough an infusion of the leaves of a small shrub, the "Sinu Mataiavi" (*Wikstraemia Indica*), is used, and there is no doubt that it has a certain expectorant value; while the expressed juice of the scraped inner bark of the same shrub is used as a stimulant for slowly healing wounds.

The purgative value of hibiscus is well known to natives in other parts of the world as well as Fiji; but there is in addition a local tree, the "Vasa" or "Rewa" (*Cerbera lactaria*), whose properties in that direction are even better, and decoctions of which are extensively indulged in.

THE HUGE LEAF OF THE VIA.

The pepsin found in the "Oleti" (*Papaya Carica*) is of proved scientific worth, and the fruit is one of the few that the native is really fond of. Although ready to eat almost anything that comes his way he seldom takes fruit if any other form of food is offered instead. If he gets a headache he makes an infusion of the scented leaves of the "Laca" a small shrub (*Plectranthus Forsteri*), and at the same time binds a circlet of "Voivoi" leaves, or any sort of fillet, tightly round his head. "Thrush" is quite a common complaint among Fijian children, and the juice of the scraped "Dani Dani" bark (*Panax fruticosum*) is used with great faith for this ailment. As a general antiseptic, however, the "Kura" (*Morinda citrifolia*) probably takes pride of place. The leaves of this tree are heated and then squeezed on to festering wounds or ulcers, more leaves being afterwards bound over the place as a sort of dressing. One may mention incidentally here that either banana leaves or the huge leaf of the "Via" will, when heated, form an absolutely waterproof material, and a useful substitute for jaconet.

*(To be continued.)*

## Figures and Tables

**Figure f1:**
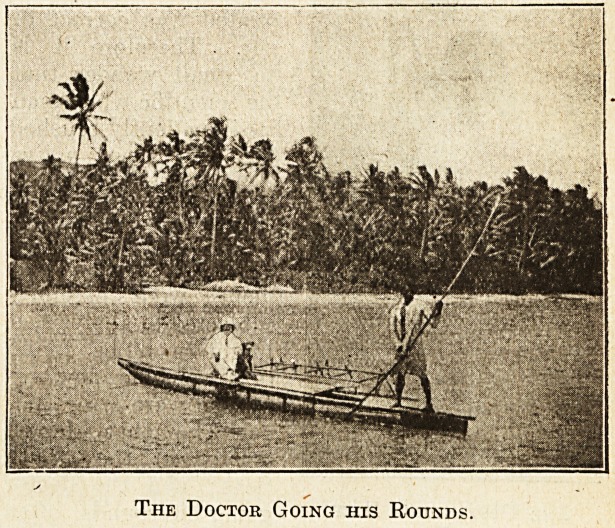


**Figure f2:**
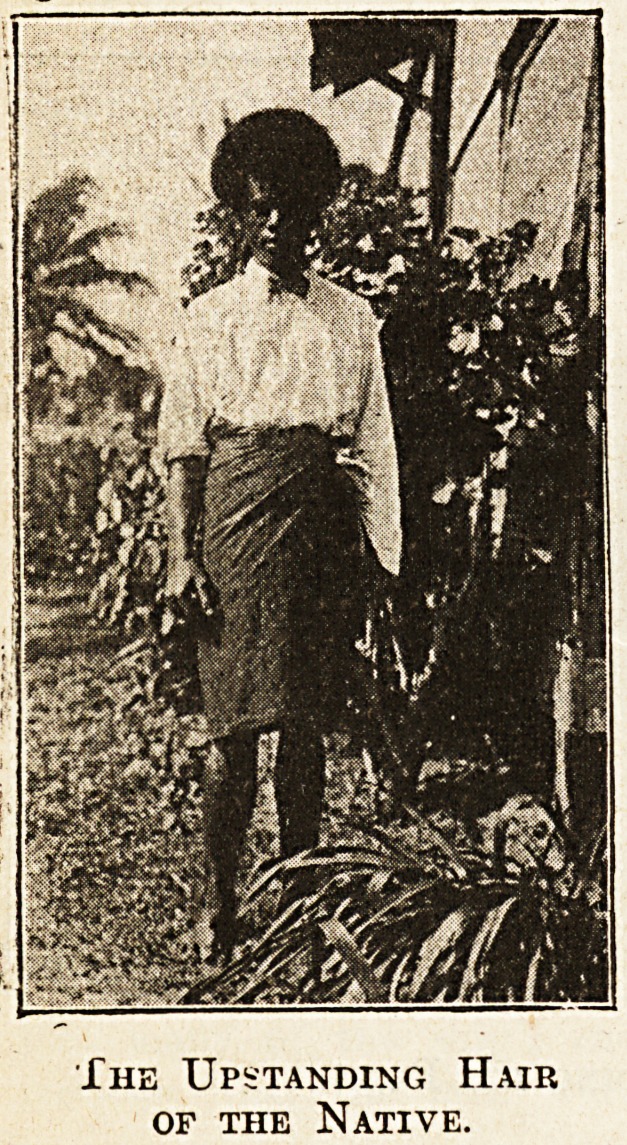


**Figure f3:**